# Development and validation of the self-administered Fibromyalgia Assessment Status: a disease-specific composite measure for evaluating treatment effect

**DOI:** 10.1186/ar2792

**Published:** 2009-08-18

**Authors:** Fausto Salaffi, Piercarlo Sarzi-Puttini, Rita Girolimetti, Stefania Gasparini, Fabiola Atzeni, Walter Grassi

**Affiliations:** 1 Department of Rheumatology, Polytechnic University of the Marche Medical School, Via dei Colli 52, 60035 Jesi (Ancona), Italy; 2 Rheumatology Unit, L. Sacco University Hospital, Via G.B. Grassi 74, 20127 Milan, Italy

## Abstract

**Introduction:**

The Fibromyalgia Impact Questionnaire (FIQ) is a composite disease-specific measure validated for fibromyalgia (FM), but it is rarely used in clinical practice. The objective was to develop and analyse the psychometric properties of a new composite disease-specific index (Fibromyalgia Assessment Status, FAS), a simple self-administered index that combines a patient's assessment of fatigue, sleep disturbances and pain evaluated on the basis of the 16 non-articular sites listed on the Self-Assessment Pain Scale (SAPS) in a single measure (range 0 to 10).

**Methods:**

The FAS index was constructed using a traditional development strategy, and its psychometric properties were tested in 226 FM patients (209 women, 17 men); whose disease-related characteristics were assessed by means of an 11-numbered circular numerical rating scale (NRS) for pain, fatigue, sleep disturbances and general health (GH), the tender point score (TPS), the SAPS, the FIQ, and the SF-36. A group of 226 rheumatoid arthritis (RA) patients was used for comparative purposes. Of the 179 FM patients who entered the follow-up study, 152 completed the three-month period and were included in the responsiveness analyses. One hundred and fifty-four patients repeated the FAS questionnaire after an interval of one week, and its test/re-test reliability was calculated. Responsiveness was evaluated on the basis of effect size and the standardised response mean.

**Results:**

The FAS index fulfilled the established criteria for validity, reliability and responsiveness. Factor analysis showed that SAPS and fatigue contributed most, and respectively explained 47.4% and 31.2% of the variance; sleep explained 21.3%. Testing for internal consistency showed that Cronbach's alpha was 0.781, thus indicating a high level of reliability. As expected, closer significant correlations were found when FAS was compared with total FIQ (rho = 0.347; *P *< 0.0001) and the FIQ subscales, particularly job ability, tiredness, fatigue and pain (all *P *< 0.0001), but the correlation between FAS and the mental component summary scale score (MCS) of the SF-36 (rho = -0.531; *P *< 0.0001) was particularly interesting. Test/re-test reliability was satisfactory. The FAS showed the greatest effect size. The magnitude of the responsiveness measures was statistically different between FAS (0.889) and the FIQ (0.781) (*P *= 0.038), and between the SF-36 MCS (0.434) and the SF-36 physical component summary scale score (PCS) (0.321) (*P *< 0.01).

**Conclusions:**

The self-administered FAS is a reliable, valid and responsive disease-specific composite measure for assessing treatment effect in patients with FM.

## Introduction

Fibromyalgia syndrome (FM) is a chronic multi-symptom disease [1-3], with pain as possibly its most important symptom. It affects approximately 2 to 3% of the general population, and more than 90% of the patients are female [4,5].

FM encompasses many symptoms, including fatigue, sleep disturbances, psychological and cognitive alterations, headache, migraine, variable bowel habits, diffuse abdominal pain, and urinary frequency [1-3], which is why studies have used a wide variety of outcome measures and assessment instruments. However, outcome measures borrowed from clinical research into pain, rheumatology, neurology, and psychiatry can only distinguish treatment responses in specific symptom domains, as has recently been highlighted by a systematic review of FM clinical trials [6]. When evaluating the effectiveness of FM therapy, it is important to be able to assess its impact on all of the domains considered important by clinicians and patients [7,8], and the OMERACT (Outcome Measures in Rheumatology) Fibromyalgia Syndrome Workshop has recently completed an attempt to include the patient perspective in identifying and prioritising such domains using focus groups and Delphi exercises [1,8,9].

Given the multifaceted nature of FM and the new therapies currently being tested [1-3], there is a need to refine these measures further to develop a reliable and valid composite patient-reported outcome (PRO) response measure that more accurately assesses treatment effects [1]. The validity and usefulness of PRO data in evaluating and monitoring patients with rheumatic conditions have been clearly documented [10,11]. PROs include physical function or disability, pain, general health status, side effects, medical costs and other factors, and instruments for measuring PROs are easier to administer and less expensive than physician-observed disease activity and process measures.

A composite disease-specific measure has been validated for FM. The Fibromyalgia Impact Questionnaire (FIQ), which was developed by Burckhardt and colleagues [12], consists of questions and visual analogue scales regarding functional disability, ability to have a job, pain intensity, sleep function, stiffness, anxiety, depression, and the overall sense of well-being. It has been shown to have a credible construct validity and reliable test/retest characteristics, and is sensitive in identifying therapeutic changes [13]. However, it is rarely used in clinical practice for a number of reasons, including its apparent lack of relevance to clinicians and their unfamiliarity with it. However, the most important reason for its lack of use seems to be the perceived difficulty in administering and scoring it. Other problems have been noted with the FIQ, including that it may underestimate disease impact and inadequately measure treatment effect in patients with mild symptoms; furthermore, it has not been validated in men [13].

The aim of this study was to develop and analyse the psychometric properties of a new composite disease-specific index for evaluating patients with FM, Fibromyalgia Assessment Status (FAS), which includes domains/items considered relevant by patients and doctors.

## Materials and methods

### Development of FAS

The development of a self-administered evaluation instrument usually follows a series of major steps: a) the identification of a specific patient population; b) the identification of important efficacy domains; c) item reduction; and d) a validation study to prove determination, reliability, validity, and responsiveness [14-16]. The process therefore begins with the development of an outcome domain pool and ends with one or more validation studies to establish test/retest reliability, construct validity, and responsiveness.

### Population identification

The aim of this study was to evaluate the disease-specific symptoms of patients who satisfy the 1990 American College of Rheumatology (ACR) classification criteria for FM [17]. Subjects with a diagnosis of anything other than chronic musculoskeletal pain conditions were excluded, as were those with medical comorbidities that would prevent them from participating fully in the study procedures (e.g. terminal conditions such as end-stage renal disease, heart failure, or malignancy), alcohol abusers, or subjects with major cognitive deficits or psychiatric symptoms that would preclude them from completing the questionnaire.

The study was approved by the Ethics Committees of the Polytechnic University of the Marche Medical School, and the Sacco University Hospital, and all of the patients gave their informed consent.

### Identification of important efficacy domains

This is considered the most important step in the development of a disease-specific evaluation instrument. The items were generated in two phases [14,18]. The first consisted of a review of the literature in order to identify the outcome measures adopted in FM clinical trials and the instruments used to assess them. The publications were retrieved by means of a comprehensive, computer-aided search of the Cochrane Central Register of Controlled Trials, MEDLINE, CINAHL, EMBASE, and PSYCINFO up to December 2008. A specific search strategy was developed for each database using the Cochrane methodological filter for randomised controlled trials and MESH keywords, and other relevant terms such as 'fibromyalgia', 'chronic pain syndrome', 'health status', 'multidisciplinary', 'patient care team', 'back pain', all of which were exploded when necessary. A manual search of the bibliographies of trials was also undertaken in order to check that all of the published trials had been identified. The search strategy led to the retrieval of 5431 articles, of which 409 were selected on the basis of their titles, abstracts and keywords. After reading all of these abstracts, 134 full-text versions of the articles were obtained, of which 41 were finally chosen.

### Domain reduction

The need for domain reduction was driven by the impossible task of carrying a large number of redundant outcome domains through the subsequent validation study. It was therefore decided to retain the 10 to 12 outcome domains that were the most important to patients and representative of their health status. In a first step, 20 potentially assessable domains in FM were reviewed for relevance by a panel of 47 experts (21 rheumatologists, 5 orthopedic surgeons, 9 physiatricians, 3 algologists, 5 psychiatrists and 3 gynecologists) using Lynn's process for content validation [19].

The second and most important step involved interviewing 87 FM patients (77 females and 10 males) attending the Rheumatology Units of Ancona, which were selected in such a way as to ensure that a wide spectrum of patient characteristics, disease severity and treatments would be elicited. The predominance of female subjects in the item generation sample was comparable with the approximate 7 to 8:1 ratio in published clinical trials. After signing an informed consent form, the patients underwent a semi-structured interview conducted by a research assistant with expertise in developing assessment instruments.

This quantitative phase measured the proportion of experts or patients who agreed that the items were relevant, as established by a content validity index (CVI). Lynn [19] recommended using a relevance rating scale that provides ordinal level data by means of four Likert-like choices (4: extremely relevant, extremely important; 3: very relevant, very important; 2: somewhat relevant, somewhat important; 1: irrelevant, unimportant). Only the items rated 3 and 4 constitute the actual CVI; the others should be eliminated. The CVI formula is: CVI or percentage agreement = number of experts agreeing on items rated as 3 or 4/total number of experts. The items were considered as having adequate content validity if agreement was 88% or more; those for which agreement was 70 to 87% were considered questionable; and those with an agreement of 69% or less were rejected. Tables 1 and 2 show the CVI values for the individual items as expressed by the physicians and patients.

**Table 1:** Content validity index values for the individual key domains identified by clinicians

	**Frequency**	**Mean importance**	**Frequency × importance product**
**Clinician-identified domains**			
			
1. Pain	100	3.9	390.0
2. Fatigue	99	3.7	366.3
3. Sleep quality	93	3.5	325.5
4. Patient global assessment	86	3.4	292.4
5. Physical function	84	3.3	277.2
6. Depression	80	3.2	256.0
7. Anxiety	77	3.3	254.1
8. Clinician global assessment	68	3.3	224.4
9. Quality of life	67	3.2	214.4
10. Occupational dysfunction	64	3.2	204.8
11. Social dysfunction	62	3.2	198.4
12. Cognitive impairment	57	3.2	182.4

**Table 2:** Content validity index values for the individual key domains identified by patients with fibromyalgia

	**Frequency**	**Mean importance**	**Frequency × importance product**
**Patient-identified domains**			
			
1. Pain	100	3.8	380.0
2. Fatigue	98	3.8	372.4
3. Sleep quality	91	3.7	336.7
4. Physical function	84	3.5	294.0
8. Morning stiffness	79	3.5	276.5
5. Anxiety	76	3.3	250.8
6. Depression	72	3.4	244.8
8. Memory problems	64	3.6	230.4
9. Quality of life	62	3.5	217.0
10. Occupational dysfunction	59	3.4	200.6
11. Social dysfunction	57	3.2	182.4
12 Problems with attention or concentration	53	3.1	164.3

A final three-item model (pain, fatigue, sleep disturbance) was judged to have adequate validity (93 to 100% agreement among the clinicians; 91 to 100% among the patients), and constituted the FAS index. Three items (physical function, depression, anxiety) rated at a level of questionable validity were closely examined by the panel of experts and then eliminated; the remaining four showed less than 69% agreement, and were eliminated without further consideration.

### Psychometric properties of FAS

The psychometric properties of the FAS index were studied in an additional cohort of 226 patients aged 20 to 75 years, who met the 1990 ACR classification criteria for FM [17] and gave their informed consent. This validation study was divided into two parts. The first part consisted of a cross-sectional study in which all 226 patients were asked to answer several questionnaires and were examined by a physician who assessed pain and other symptoms; 163 of these patients repeated the evaluation after an interval of one week in order to test its reliability. For purposes of comparison, we also evaluated a sample of 226 patients meeting the ACR criteria for rheumatoid arthritis (RA) [20], who were randomly matched from 469 RA patients participating in an ongoing longitudinal outcome project and reflected the age/gender-related stratification/distribution of the FM sample, and underwent the same complete clinical assessment with the fibromyalgia tender points assessment but the FIQ was not administered [12,21]. They also completed the Medical Outcomes Study Short Form-36 Health Survey (SF-36) [22,23].

The second part consisted of a three-month follow-up period during which we assessed the sensitivity of the FAS to changes in the 179 FM patients who had started a new pharmacological treatment (muscle relaxants and antidepressants were the most frequently used medications) or significantly changed the dose of their existing treatment. One hundred and fifty-two completed this part of the study; the other 27 did not attend our outpatient clinic during this time and were excluded from the analysis although retrospective data checks revealed that they experienced the same disease course. The study was performed in accordance with the principles of the Declaration of Helsinki, and the protocols were approved by our Ethics Committees.

#### Clinical assessment

The patients were administered a questionnaire including questions relating to sociodemographic data, disease-related variables and the quality of life. The sociodemographic variables were age, gender, education, marital status, and the duration of FM symptoms. Age and symptom duration were recorded in years; education was divided into three categories based on the Italian school system (1 = primary school, 2 = secondary school, and 3 = high school or university); and marital status was divided into two categories (1 = living with a partner; 0 = living alone). The assessment of comorbidities included nine specific conditions: hypertension, myocardial infarction, lower extremity arterial disease, major neurological problems, diabetes, gastrointestinal disease, chronic respiratory disease, kidney disease, and poor vision.

#### Measurements and instruments

The disease-related characteristics included a patient 11-numbered circular numerical rating scale (NRS) for pain [24], fatigue, sleep disturbances, and general health (GH), the tender point score (TPS), and the Self-assessment Pain Scale (SAPS).

The NRS questions were: 'Please choose a number between 0 and 10 that best describes the average level of pain you have experienced in the past week (0 = no pain; 10 = pain as bad as it can be)'; 'What number between 0 and 10 best describes the average level of fatigue you have experienced in the past week (0 = no fatigue; 10 = fatigue as bad as it can be)?'; 'How much of a problem has sleep been in the past week (0 = no problem; 10 = severe problem)?'; and 'How would you describe your general health over the past week (0 = very good; 10 = very bad)?'.

The tender point examination was carried out by applying the same manual finger pressure with a force of 4 kg (until blanching of the fingernail bed) to each of nine paired anatomical locations The 18 FM tender point sites were: bilateral occiput, low cervical, trapezius, supraspinatus, second rib, lateral epicondyle, gluteal, greater trochanter, and knee [1,17]. For a tender point to be considered 'positive', the patient had to state that the palpation was painful. Regular consensus meetings concerning tender point assessments are part of our routine quality control programme in order to avoid high between-physician variations, but no formal agreement analysis was made for the purpose of this study. The TPS was the total number of tender points.

**Figure 1:**
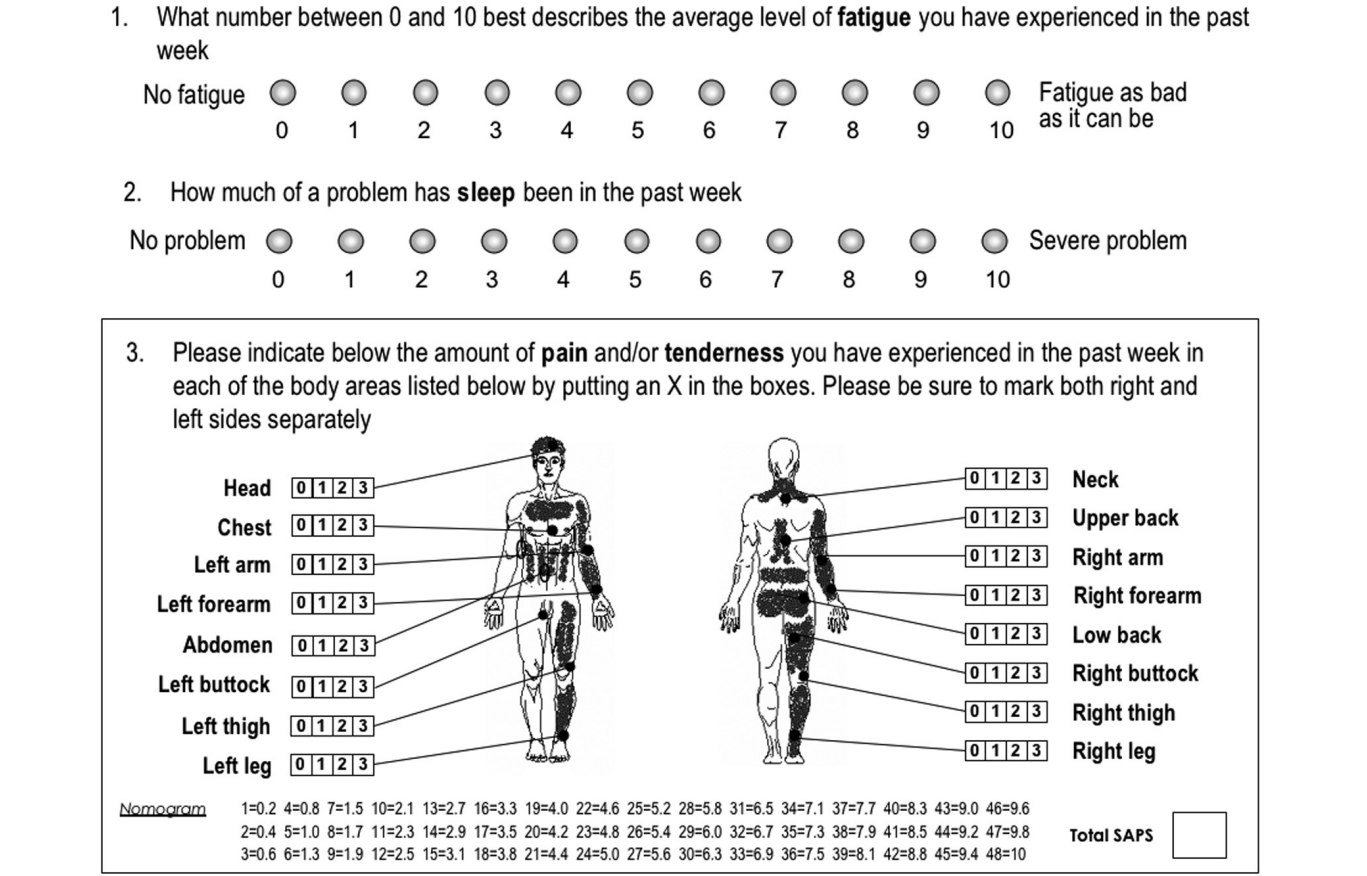
The self-administered Fibromyalgia Assessment Status (FAS).

The SAPS considered the pain 'experienced during the past week' in 16 non-articular sites as follows: 'Please indicate below the amount of pain and/or tenderness you have experienced in the last seven days in each of the body areas listed below by putting an X in the boxes (see Figure 1). Please be sure to mark both right and left sides separately'. Below these instructions, a series of site descriptions were followed by four boxes labelled 0 = none, 1 = mild, 2 = moderate, and 3 = severe. *The scale *scores range from 0 to 48 but, in order to integrate them into one scale they were transformed to a scale of 0 to 10. We then calculated the FAS index, which is a short and easy to complete self-administered index combining a set of questions relating to non-articular pain (SAPS range 0 to 10), fatigue (range 0 to 10), and the quality of sleep (range 0 to 10) that provides a single composite measure of disease activity ranging from 0 to 10. The final score is calculated by adding the three sub-scores and dividing the result by three. All three measures are printed on one side of one page for rapid review, and scored by a health professional without the need for a ruler, calculator, computer, or website (Figure 1).

Two quality of life questionnaires were also administered: the specific self-administered FIQ [12] and the generic SF-36 [22]. The FIQ consists of 10 sub-items: the first includes 11 questions concern physical functioning, and each is rated using a four-point Likert scale; items 2 and 3 ask the patient to mark the number of days they felt well and the number of days they were unable to work (including housework) because of FM symptoms; and items 4 to 10 are horizontal linear 10-increment scales by means of which the patients rate the number of days on which they felt good, the number of working days missed, ability to do their job, pain, fatigue, morning tiredness, stiffness, anxiety, and depression [12]. Each item has a maximum score of 10, and so the highest possible score is 100 (the higher the score, the greater the impact of the syndrome on the person). The Italian version of the FIQ has been previously validated [21].

The SF-36 is a general health questionnaire divided into eight scales, each of which measures a different aspect of health [22]. The sub-scale scores are then transformed into a 0 to 100 scale using a scoring algorithm, with higher scores indicating a better quality of life. The SF-36 has been validated for use in Italy [23], and can be completed by most people within 15 minutes. The creators of the SF-36 have also developed algorithms to calculate two psychometrically based summary measures: the physical component summary scale score (PCS) and the mental component summary scale score (MCS) [25].

### Statistical analysis

Following standard guidelines for evaluating the properties of composite measures, we tested the construct validity, test/retest reliability, and responsiveness of the FAS index. Construct validity was investigated in three ways. We first explored the underlying component structure of the items by means of exploratory factor analysis (principal component analysis) using principal axis extraction and the varimax rotation method, which maximises the independence of the factors. Principal component analysis was chosen in order to reveal the dimensionality of the score in the patient cohorts and investigate factor loading. An eigenvalue criterion of 1.0 was used to select the factors, and the results are given in terms of the percentage variance in the scale score explained by the principal factor. As an indicator of internal consistency reliability, we calculated Cronbach values (achievable values range from 0, indicating no internal consistency, to 1, indicating identical results), and Cronbach alpha values of more than 0.7 are commonly considered markers of a high degree of reliability. We then examined convergent validity by correlating the scores of the index with the other measures used in the study (the score of a given scale is expected to converge with those of other instruments targeting the same construct, and deviate from those of other instruments assessing a different construct) and quantifying these relationships using Spearman's rho correlation coefficients. Thirdly, in order to investigate the possible influence of patient characteristics such as age, marital status, education, and the number of comorbidities, the associations between these and the FAS index were quantified using Spearman's correlation coefficients, Wilcoxon's rank sum test and Kruskal-Wallis one-way analysis of variance, with the differences being considered significant when the *P *value was less than 0.05. Discriminant validity was assessed by means of receiver operating characteristic (ROC) curves and by comparing the ability of the FAS index to distinguish the FM and RA patients participating in the study. ROC curves were plotted for each model in order to determine its area under the curve (AUC), sensitivity and specificity, and then used to compute the optimal cut-off value corresponding to the maximum sum of sensitivity and specificity.

Wilcoxon's signed rank test and Fisher's exact test were respectively used for the between-group comparisons of all continuous and categorical variables. Test/retest reliability embraces the concept that the repeated administration of a measurement instrument to stable subjects will yield the same results. After a one-week interval, the patients were asked by the same investigator to repeat all of the clinical measures without having access to any of the previous ratings. As it was possible for a patient's condition to change during this period, the subjects were concurrently administered a 'transitional' global rating of change questionnaire in which they were asked: 'How is your health now in comparison with when you completed the health status questionnaire one week ago?'. The possible response options were 'much better', 'slightly better', 'no change', 'slightly worse', or 'much worse'. The subjects who reported no change were considered stable and those who reported a change were removed from the analysis.

Wilcoxon's signed rank test and concordance correlation coefficients (CCC) with 95% confidence intervals (CI) of the mean values were used to check for any significant systematic differences in test/retest administration [26]. The agreements between scores were also illustrated by Bland and Altman plots, with a level of statistical significance of *P *< 0.05 (two-sided). Responsiveness was tested using effect size (ES) and standardised response means (SRMs) [27,28]. The change due to intervention was assessed using Wilcoxon's non-parametric signed rank test, which has the advantage of being robust to distributional assumptions. The chosen level of significance was α = 0.05. ES is calculated as the mean change in score from baseline divided by the standard deviation of the baseline scores, whereas SRM is the mean change in score between assessments divided by the standard deviation of these changes. The 'modified jack-knife test' was used to test whether the difference between two responsiveness measures was statistically significant. The data were processed and analysed using SPSS software (Windows release 11.0; SPSS Inc., Chicago, IL, USA), and MedCalc Software^® ^(Windows release 11.0.0, Mariakerke, Belgium).

## Results

### Study participants

The study involved 226 FM patients (209 women and 17 men) with a mean age of 52.1 ± 10.8 years (range 20 to 75), a mean duration of symptoms of 10.5 ± 9.7 years (range 1 to 28), a mean TPS of 15.1 ± 2.4 (range 11 to 18), and a mean pain intensity of 6.8 ± 2.1 (range 2 to 10) as measured using an 11-numbered circular NRS. Their educational level was generally low: 41.2% had only attended a primary school, and only 17.9% had attended a high school. Sixty-five percent were living with a partner. The most frequently reported comorbid conditions were cardiovascular disorders (20.1%), metabolic disorders (12.7%), chronic pulmonary disease (10.2%), and gastrointestinal diseases (7.3%): 29.1% of the patients reported one, and 19% two or more (range 2 to 5). The FM patients reported significantly greater levels of fatigue (7.4 ± 4.3; P < 0.001) and sleep disturbance (6.9 ± 4.2; P < 0.001) than the RA patients (206 women, 20 men), who were similar in terms of age (mean age 56.1 ± 11.4 years, range 34 to 87), education level and marital status. The arithmetic mean (standard deviation) of FAS was 6.34 (1.61) and the 95% CI of the mean was 6.09 to 6.49.

### Validity analysis

**Figure 2:**
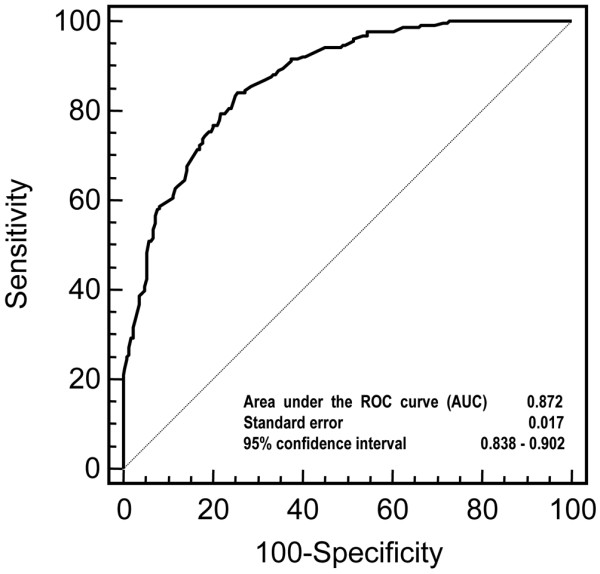
Fibromyalgia Assessment Status receiver operating characteristic curve. The results of the sensitivity and specificity analyses of various cut-off points for the composite index are summarised. We analysed the ability of Fibromyalgia Assessment Status to identify patient populations: the greater the area under the curve (AUC), or the further the distance to the 'change line', the better its discriminant power. ROC = receiver operating characteristic.

The construct validity of the FAS index was examined in terms of convergence and discriminant validity. Factor analysis showed that the index constitutes a monocomponent measure in FM. SAPS and fatigue contributed most, and respectively explained 47.46% and 31.23% of the explained variance; sleep explained 21.29%. When testing internal consistency reliability, we found that Cronbach's alpha was 0.781, which indicates a high degree of reliability. As expected, the FAS index had more significant correlations with total FIQ (rho = 0.347; *P *< 0.0001) and the FIQ sub-scales, particularly job ability fatigue (rho = 0.534; *P *< 0.0001), fatigue (rho = 0.379; *P *< 0.0001), morning tiredness (rho = 0.309; *P *< 0.0001), and pain (rho = 0.303; *P *< 0.0001) (convergent construct validity; Table 3). There were negative correlations with the SF-36 as higher SF-36 scores indicate more and higher FAS scores less well-being: the correlation between FAS and SF-36 MCS (rho = -0.531; *P *< 0.0001; Table 4) was particularly interesting, but the correlations with the SF-36 sub-scales and summary measures were not as close as those between FAS and the FIQ. The three component variables of FAS correlated with each other moderately to highly, with the closest correlation between NRS-fatigue and NRS-sleep (rho = 0.568; *P *< 0.0001). There were also close correlations between the TPS and FAS (rho = 0.391; *P *< 0.0001), between the SAPS and the SF-36 MCS (rho = -0.297; *P *< 0.0001), and between the TPS and the SF-36 MCS (rho = -0.373; *P *< 0.0001). Women tended to have higher FAS values than men (Wilcoxon's test: W = -2.19; *P *= 0.022), but there were no significant gender or age-related differences (four age-groups ranging from 20 to 34 years to 75 years). The respondents with a low educational level were more often classified as having high levels of disease activity, and stratification into three categories confirmed that increasing education was associated with lower FAS values: primary school = 7.2 ± 1.8; secondary school = 6.3 ± 1.5; high school/university = 5.5 ± 1.6; Kruskal-Wallis test: *P *< 0.002). Furthermore, the patients with comorbid conditions had worse disease activity scores (Kruskal-Wallis test: *P *< 0.004). The ROC curve used to discriminate FM and RA patients is shown in Figure 2. The discriminating power of the FAS index was good, with an AUC of 0.872 (95% CI: 0.838 to 0.902). Each point of the ROC curve represents the true-positive (or sensitivity) and false-positive ratios (or 1-specificity) of a particular cut-off value, and may help in selecting the optimal cut-off value for a new scale: i.e. assuming an optimal FAS cut-off value of 5.7, sensitivity was 78.8% and specificity 74.5%. Higher cut-off values led to greater sensitivity but lower specificity, whereas a cut-off value of 4.6 gave a sensitivity of 58.7% with a specificity of 91.9%.

**Table 3:** Convergent construct validity analysis: correlation matrix of overall Fibromyalgia Assessment Status scores and their components *vs *the Fibromyalgia Impact Questionnaire dimensions

			**Physical functioning**	**Number of days felt good**	**Number of working days missed**	**Job ability**	**Pain**	**Fatigue**	**Tiredness**	**Stiffness**	**Anxiety**	**Depression**	**Total FIQ**
**Spearman's rho**	**SAPS**	Correlation coefficient	0.217(**)	0.195(**)	0.191(**)	0.145 (*)	0.271(**)	0.136(*)	0.147(*)	0.136(*)	0.260(**)	0.212(**)	0.193(**)
	**FATIGUE**	Correlation coefficient	0.571(**)	0.482(**)	0.568(**)	0.568 (**)	0.663(**)	1.000(**)	0.568(**)	0.556(**)	0.411(**)	0.257(**)	0.804(**)
	**SLEEP**	Correlation coefficient	0.424(**)	0.259(**)	0.397(**)	0.397 (**)	0.391(**)	0.568(**)	1.000(**)	0.379(**)	0.326(**)	0.256(**)	0.618(**)
	**FAS**	Correlation coefficient	0.294(**)	0.251(**)	0.257(**)	0.534 (**)	0.303(**)	0.379(**)	0.309 (**)	0.147(*)	0.255(**)	0.217(**)	0.347(**)

** Correlation significant at 0.001 level (2-tailed).

* Correlation significant at 0.01 level (2-tailed).

FAS = Fibromyalgia Assessment Status; SAPS = Self-Assessment Pain Scale.

**Table 4:** Convergent construct validity analysis: correlation matrix of overall FAS scores and their components *vs *the SF-36 dimensions

			**Medical outcomes SF-36 health survey**									
			
			**PF**	**RF**	**BP**	**GH**	**VT**	**SF**	**RE**	**MH**	**PCS**	**MCS**
**Spearman's rho**	**SAPS**	*Correlation coefficient*	-0.142 (*)	-0.141 (*)	-0.214 (**)	-0.187 (**)	-0.175 (*)	-0.213 (**)	-0.242 (**)	-0.269 (**)	-0.139 (*)	-0.297 (**)
	**FATIGUE**	*Correlation coefficient*	-0.143 (*)	-0.297 (**)	-0.451 (**)	-0.189 (**)	-0.670 (**)	-0.270 (**)	-0.327 (**)	-0.306 (**)	-0.342 (**)	-0.401 (**)
	**SLEEP**	*Correlation coefficient*	0.148 (*)	0.139 (*)	-0.246 (**)	-0.213 (**)	-0.518 (**)	0.141 (*)	-0.276 (**)	-0.288 (**)	0.154 (*)	-0.401 (**)
	**FAS**	*Correlation coefficient*	-0.138 (*)	-0.157 (*)	-0.336 (**)	-0.267 (*)	-0.593 (**)	-0.225 (**)	-0.318 (**)	-0.350 (**)	-0.240 (**)	-0.531 (**)

** Correlation significant at 0.001 level (2-tailed).

* Correlation significant at 0.01 level (2-tailed).

BP = bodily pain; FAS = Fibromyalgia Assessment Status; GH = perceived general health; MCS = mental component scale summary score; MH = mental health; PCS = physical component scale summary score; PF = physical functioning; RE = role function/emotional aspect; RF = role function/physical aspect; SAPS = Self-Assessment Pain Scale; SF = social functioning; SF-36 = Short Form 36 Health Survey; VT = vitality.

### Reliability analysis

**Figure 3:**
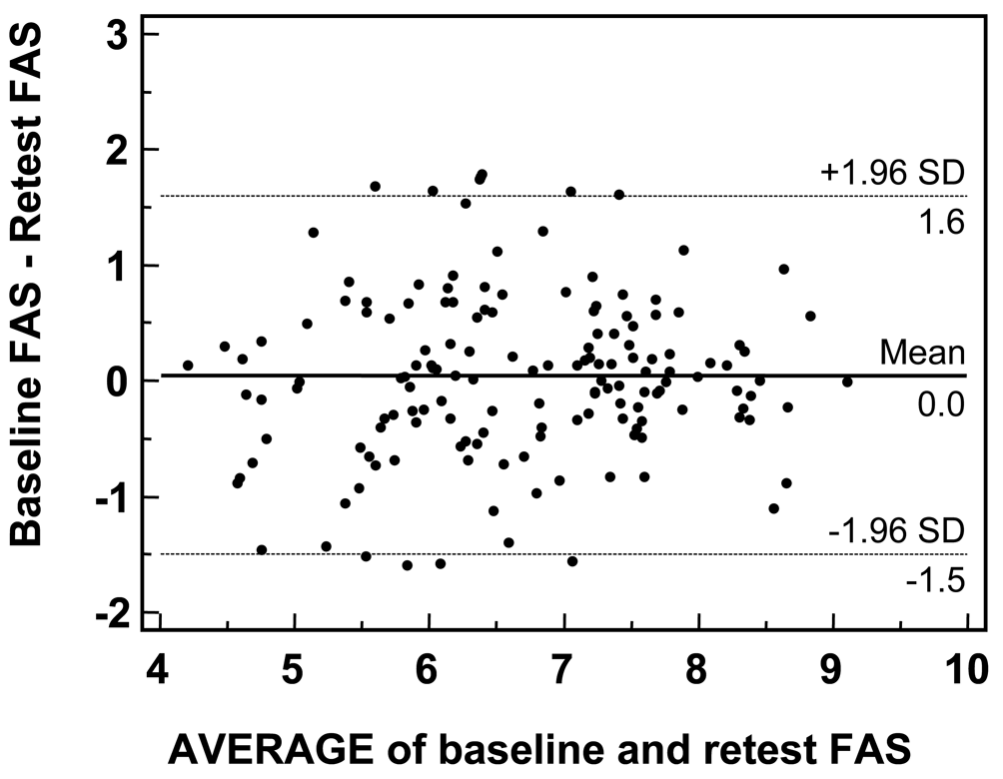
Bland and Altman plot of repeatability, with the differences in Fibromyalgia Assessment Status values plotted against average values. Ninety-five percent of the differences against the means were less than two standard deviations (SD; dotted lines).

The reliability of the FAS index was evaluated in 163 patients over a one-week period. Nine subjects were excluded because they reported a change in health between the test and retest. For the remaining 154 subjects, the mean interval was 6.5 ± 1.5 days. The CCC of the index was 0.853 (95% CI 0.803 to 0.858). Figure 3 shows the Bland and Altman plot of repeatability: 95% of the differences against the means were less than two standard deviations.

### Responsiveness analysis

Table 5 shows the results of Wilcoxon's test, and the ES and SMR statistics for the individual measures, FAS and the questionnaires in the FM sample. On the basis of the conventional interpretation of ES, all of the measures improved significantly during the three-month follow-up period. The greatest improvements were found for FAS, and the smallest for TPS and the SF-36 PCS and MCS component summary scores. Within the generic SF-36 measure, the mental component improved more than the physical component. The magnitude of the responsiveness measures (assessed by means of the individual ES) was statistically different between FAS (ES = 0.889) and the FIQ (ES = 0.781; *P *= 0.038), and between the SF-36 MCS (ES = 0.434) and the SF-36 PCS (ES = 0.321; *P *< 0.01). SRM tended to be lower than the ES, but followed a similar pattern.

**Table 5:** Indices of responsiveness after three months of follow-up in fibromyalgia patients

	**Mean change**	**Wilcoxon's test**	**P value**	**Effect size**	**Standardised response mean**
Pain	5.141	5.653	< 0.0001	0.535	0.606
Fatigue	2.221	8.112	< 0.0001	0.787	0.778
Sleep	1.682	5.765	0.0008	0.698	0.518
Stiffness	1.864	5.785	0.0006	0.627	0.536
GH	0.941	6.882	< 0.0001	0.581	0.444
TPS	0.453	2.154	0.0312	0.191	0.151
SAPS	1.312	9.911	< 0.0001	0.713	0.722
FAS	1.431	10.015	< 0.0001	0.889	0.831
FIQ	14.194	8.184	< 0.0001	0.781	0.819
SF-36 PCS	2.594	-3.366	0.0006	0.321	0.285
SF-36 MCS	5.029	-4.412	< 0.0001	0.434	0.384

The greatest improvements were in Fibromyalgia Assessment Status (FAS), and the smallest in tender point score (TPS) and Short Form 36 Health Survey (SF-36) physical component scale summary score (PCS) and mental component scale summary score (MCS). FIQ = Fibromyalgia Impact Questionnaire; GH = perceived general health; SAPS = Self-Assessment Pain Scale.

## Discussion

One of the main problems in developing an efficacy claim for FM is the lack of consensus concerning the response criteria that should be used as primary outcome measures in clinical trials, which means that further work is necessary to refine and validate the existing measures, and develop new composite measures or response criteria that better address the multidimensional nature of the syndrome and can also be used in everyday clinical care [29-31]. The Disease Activity Score (DAS) used in RA is a good example of an appropriate index, because it has been shown to perform well in clinical research and has also been implemented and accepted in clinical practice even though the DAS algorithm is rather complex [32,33].

In general, and referring to the OMERACT initiative, such indices should be truthful, discriminant, responsive, and feasible [34]. To meet these aims, two approaches were combined. First of all, the domains considered to be most relevant were first consensually selected by experts and patients in order to obtain a high face validity. Secondly, and following standard guidelines for evaluating the properties of composite measures, we tested the construct validity, test/retest reliability and responsiveness of the FAS index.

In line with the methodology adopted by OMERACT [2], we conducted a Delphi exercise involving a panel of 47 experts to develop consensus on a prioritised list of key domains of the FM syndrome that should be addressed in clinical trials. A final three-item model (pain, fatigue, sleep disturbance) was judged to have adequate validity (93 to 100% agreement among the clinicians; 91 to 100% among the patients), and constituted the FAS index. It is interesting to note that patients rated stiffness much higher than clinicians, as also occurred during the OMERACT workshop consensus voting [2].

The data showed that the FAS index had good psychometric properties as a multidimensional PRO instrument for FM that is consistent with the recommendations of the OMERACT Fibromyalgia Syndrome Workshop [1,9] and the IMMPACT group (Initiative on Methods, Measurement, and Pain Assessment in Clinical Trials) [35].

It does not include data concerning psychological distress, change in status, ability to do a job, morning stiffness, or the other constructs included in the FIQ [12]. The several reasons for the lack of use and perceived difficulty in administering and scoring the FIQ [13] persuaded us to develop simpler and more easily scored patient questionnaires for use in standard clinical care, which can be scanned by a clinician in 10 to 20 seconds or less, scored in less than 30 seconds, and which provide information concerning the patients' perceived widespread pain, average level of fatigue, and sleep disturbance all on one side of one page.

When testing its internal construct validity, factor analysis showed that the FAS index constitutes a monocomponent measure in FM, in which SAPS (which represents the patients' perception of widespread pain) accounts for 47.67% of the explained variance, fatigue (the patients' average level of fatigue during the previous week) 31.23%, and sleep disturbance 21.29%. This is in line with the findings of Staud and colleagues, who demonstrated that peripheral factors (maximum average local pain and the markers of painful body areas) predict most of the variance in overall clinical pain, and suggested that pain input from peripheral tissues is clinically relevant [36]. The SAPS questionnaire is one approach to analysing the extent of body pain and evaluates pain intensity and its non-articular regional speed.

The number of peripheral pain areas and peripheral pain intensity are better predictors of overall FM pain than the TPS, and this seems to indicate their pathogenetic relevance [37] and may explain why SAPS has good discriminant power. In comparison with RA, FM is mainly characterised by the different nature of its pain. Simms and colleagues have shown that a pain visual analogue scale is less discriminating than pain measured with its regional component [30], and the fact that SAPS integrates pain distribution and severity makes it a very specific instrument for FM. In addition to being the cardinal symptom of FM, pain is also one of the strongest predictors of fatigue. Individuals with higher average pain levels report greater fatigue, and daily increases in pain are related to daily increases in fatigue, including those relating to the following day [38].

The validity of the FAS index was also supported by its significant correlations with the TPS, the FIQ and its sub-scales, and other self-reported generic measures such as physical disability on the SF-36 PCS and emotional state on the SF-36 MCS [39]. The correlations between FAS and SF-36 MCS, and between FAS and the anxiety/depression sub-scales of the FIQ (all *P *< 0.0001) are particularly interesting. A number of studies have highlighted the important contribution of local pain and negative pain affect to clinical pain intensity, and this underlines the multidimensional nature of clinical pain intensity in FM patients [40,41], as well as the general population [42-44]. Like other self-report instruments, the FAS index is sensitive to psychosocial factors, which contribute to the pain and physical impairment reported by patients. Furthermore, negative mood also seems to contribute to the persistence of chronic widespread pain [45,46].

If emotional state markedly influences a patient's perception of pain and physical health status, the resulting random measurement error would restrict the validity of the FAS index or other self-report questionnaires to relatively large studies but, when we examined the affective correlates of fatigue and sleep abnormalities, we found strong evidence that they were also associated with negative affect (as shown by the anxiety/depression sub-scales of the FIQ). These findings are only partially consistent with previous studies of individual differences in fatigue [47,48], although it has been found that FM patients who report greater average fatigue also report more sleep problems and higher levels of negative affect [38].

We also investigated the relations between FAS and the main sociodemographic characteristics and comorbidities, and our data show that there were no significant gender or age-related differences, whereas respondents with a low educational level were more often classified as having a high degree of disease activity. It has been reported that years of formal education are a risk factor for the presence of chronic pain in the community [49,50]. Furthermore, Callahan and colleagues [51] found education to be related to pain severity as measured by a simple visual analogue score. The mechanism by which education influences pain severity is unclear, but it may be related to enhanced self-efficacy and a sense of control allowing a patient to take advantage of a greater number of pain-reducing modalities. Furthermore, self-reported chronic pain or physical dysfunctions may not only be due to musculoskeletal health, but also to other prevalent causes of restricted mobility such as cardiovascular and respiratory disorders, and our patients with comorbidities had worse disease activity scores (*P *< 0.004). Bombardier and colleagues [52] found that SF-36 pain and physical function scores decreased as the number of comorbidity factors increased. The pattern of the association of chronic pain with sociodemographic factors is interesting, and supports the findings of previous studies of chronic pain [44,46,49,50], but it is not clear from our cross-sectional research whether they reflect causes or effects. Wolfe and Rasker [53] found that higher scores on the Symptom Intensity scale are associated with more severe medical illness, greater mortality and sociodemographic disadvantage, and these factors also seem to play a role in the development of FM-like symptoms and symptom intensification. Our study equally cannot determine whether all of the demonstrated impaired well-being was directly attributable to the presence of chronic pain (because of the possibility of confounding variables such as comorbidity or the fact that pain may be a secondary symptom of another condition such as ischemic heart or digestive diseases) or chronic peripheral neuropathic pain.

One further limitation that has to be considered is our non-randomised primary care sample. It can be assumed that the motivation of patients who volunteer to take part in a study is different from that of a random population, and they may have a tendency to exaggerate self-perceived severity.

The repeatability of the FAS index was excellent, as shown by the CCC, and the Bland-Altman plots showed that 95% of the differences against the means were less than two standard deviations. This has to be taken into account in clinical practice because the change in scores at individual level must exceed the level of random error in order to reflect a real difference in health status.

The responsiveness of the FAS index was confirmed by the ES and SRM statistics, whose conventional interpretation showed that all of the measures had significantly improved three months after starting treatment, with the greatest improvements being found for FAS and the FIQ, and the smallest for the TPS and the SF-36 PCS and MCS scores. The mental component of the generic SF-36 measure improved more than the physical component. The SRMs generally yielded somewhat smaller numbers but did not change the interpretation of the data. One final disadvantage of this study is that no placebo group was included as a control, and it is possible that the use of an open-label design may have increased the differences before and after treatment.

## Conclusions

In conclusion, our findings suggest that the self-administered FAS index is a valid, reliable, and responsive composite disease-specific measure for assessing treatment effects in patients with FM that can be used in clinical trials and everyday clinical practice. As the FAS index involves the use of only one side of one page, it can be quickly reviewed by clinicians to obtain a simple overview of patient status. Furthermore, it should allow physicians to obtain reliable information concerning the course of the disease, and be sensitive enough to raise alarm in the case of deterioration. Its generalisability and usefulness in assessing treatment and long-term outcomes now need to be evaluated in broader settings.

## Abbreviations

ACR: American College of Rheumatology; AUC: area under the curve; CCC: concordance correlation coefficients; CI: confidence interval; CVI: content validity index; DAS: Disease Activity Score; ES: effect size; FAS: Fibromyalgia Assessment Status; FIQ: Fibromyalgia Impact Questionnaire; FM: Fibromyalgia; GH: general health; IMMPACT: Initiative on Methods, Measurement, and Pain Assessment in Clinical Trials; MCS: mental component summary scale score; NRS: numerical rating scale; OMERACT: Outcome Measures in Rheumatology; PRO: patient-reported outcome; PCS: component summary scale score; RA: rheumatoid arthritis; ROC: receiver operating characteristic; SAPS: Self-Assessment Pain Scale; SF-36: Short Form 36 Health Survey; SRMs: standardised response means; TPS: tender point score.

## Competing interests

The authors declare that they have no competing interests.

## Authors' contributions

FS contributed to the conception of the study, and the acquisition, analysis and interpretation of the data, and participated in drafting the manuscript. PSP contributed to the conception of the study, and acquisition, analysis and interpretation of the data, and participated in drafting the manuscript. RG participated in the analysis and interpretation of the data. SG contributed to the acquisition of the data. FA contributed to the interpretation of the data and critically reviewed the manuscript. WG provided final approval of the version to be published.

## References

[B1] Mease P (2005). Fibromyalgia syndrome: review of clinical presentation, pathogenesis, outcome measures, and treatment. J Rheumatol Suppl.

[B2] Mease P, Arnold LM, Bennett R, Boonen A, Buskila D, Carville S, Chappell A, Choy E, Clauw D, Dadabhoy D, Gendreau M, Goldenberg D, Littlejohn G, Martin S, Perera P, Russell IJ, Simon L, Spaeth M, Williams D, Crofford L (2007). Fibromyalgia syndrome. J Rheumatol.

[B3] Bennett RM, Jones J, Turk DC, Russell J, Matallana L (2007). An internet survey of 2,596 people with fibromyalgia. BMC Musculoskeletal Disorders.

[B4] Wolfe F, Ross K, Anderson J, Russell IJ, Hebert L (1995). The prevalence and characteristics of fibromyalgia in the general population. Arthritis Rheum.

[B5] Salaffi F, De Angelis R, Stancati A, Grassi W, MArche Pain; Prevalence INvestigation Group (MAPPING) study (2005). Health-related quality of life in multiple musculoskeletal conditions: a cross sectional population based epidemiological study. II. The MAPPING study. Clin Exp Rheumatol.

[B6] Carville SF, Choy EHS (2008). Systematic review of discriminating power of outcome measures used in clinical trials of fibromyalgia. J Rheumatol.

[B7] Sarzi-Puttini P, Buskila D, Carrabba M, Doria A, Atzeni F (2008). Treatment strategy in fibromyalgia syndrome: where are we now?. Semin Arthritis Rheum.

[B8] Carville SF, Arendt-Nielsen S, Bliddal H, Blotman F, Branco JC, Buskila D, Da Silva JA, Danneskiold-Samsøe B, Dincer F, Henriksson C, Henriksson KG, Kosek E, Longley K, McCarthy GM, Perrot S, Puszczewicz M, Sarzi-Puttini P, Silman A, Späth M, Choy EH, EULAR (2008). EULAR evidence-based recommendations for the management of fibromyalgia syndrome. Ann Rheum Dis.

[B9] Mease PJ, Arnold LM, Crofford LJ, Williams DA, Russell IJ, Humphrey L, Abetz L, Martin SA (2008). Identifying the clinical domains of fibromyalgia: contributions from clinician and patient Delphi exercises. Arthritis Rheum.

[B10] Pincus T, Maclean R, Yazici Y, Harrington JT (2007). Quantitative measurement of patient status in the regular care of patients with rheumatic diseases over 25 years as a continuous quality improvement activity, rather than traditional research. Clin Exp Rheumatol.

[B11] Levy G, Cheetham C, Cheatwood A, Burchette R (2007). Validation of patient-reported joint counts in reumatoid arthritis and the role of training. J Rheumatol.

[B12] Burckhardt CS, Clark SR, Bennett RM (1991). The Fibromyalgia Impact Questionnaire: development and validation. J Rheumatol.

[B13] Bennett R (2005). The Fibromyalgia Impact Questionnaire (FIQ): a review of its development, current version, operating characteristics and uses. Clin Exp Rheumatol.

[B14] Kirshner B, Guyatt GH (1985). Methodological framework for assessing health indices. J Chron Dis.

[B15] Salaffi F, Bazzichi L, Stancati A, Neri R, Cazzato M, Consensi A, Grassi W, Bombardieri S (2005). Development of a functional disability measurement tool to assess early arthritis: the Recent-Onset Arthritis Disability (ROAD) questionnaire. Clin Exp Rheumatol.

[B16] Salaffi F, Silveri F, Stancati A, Grassi W (2005). Development and validation of the osteoporosis prescreening risk assessment (OPERA) tool to facilitate identification of women likely to have low bone density. Clin Rheumatol.

[B17] Wolfe F, Smythe HA, Yunus MB, Bennett RM, Bombardier C, Goldenberg DL, Tugwell P, Campbell SM, Abeles M, Clark P, Fam AG, Farber SJ, Fiechtner JJ, Michael Franklin C, Gatter RA, Hamaty D, Lessard J, Lichtbroun AS, Masi AT, Mccain GA, John Reynolds W, Romano TJ, Russell IJ, Sheon RP (1990). The American College of Rheumatology 1990 criteria for the classification of fibromyalgia. Report of the Multicenter Criteria Committee. Arthritis Rheum.

[B18] Kirkley A, Griffin S, McClintock J, Ng L (1998). The development of a disease specific quality of life measurement tool for shoulder instability. Am J Sports Med.

[B19] Lynn MR (1986). Determination and quantification of content validity. Nurs Res.

[B20] Arnett FC, Edworthy SM, Bloch DA, McShane DJ, Fries JF, Cooper NS, Healey LA, Kaplan SR, Liang MH, Luthra HS, Medsger TA, Mitchell DM, Neustadt DH, Pinals RS, Schaller JG, Sharp JT, Wilder RL, Hunder GG (1988). The American Rheumatism Association 1987 revised criteria for the classification of rheumatoid arthritis. Arthritis Rheum.

[B21] Sarzi-Puttini P, Atzeni F, Fiorini T, Panni B, Randisi G, Turiel M, Carrabba M (2003). Validation of an Italian version of the Fibromyalgia Impact Questionnaire (FIQ-I). Clin Exp Rheumatol.

[B22] Ware J, Sherbourne CD (1992). The MOS 36-item short form health survey (SF-36). 1. Conceptual frame-work and item selection. Med Care.

[B23] Apollone G, Mosconi P (1998). The Italian SF-36 Health Survey: translation, validation and norming. J Clin Epidemiol.

[B24] Pincus T, Bergman M, Sokka T, Roth J, Swearingen C, Yazici Y (2008). Visual analog scales in formats other than a 10 centimeter horizontal line to assess pain and other clinical data. J Rheumatol.

[B25] Ware JE, Kosinski M (2001). . Interpreting SF-36 summary health measures: a response. Qual Life Res.

[B26] Lin LI-K (1989). A concordance correlation coefficient to evaluate reproducibility. Biometrics.

[B27] de Bruin AF, Diederiks JP, de Witte LP, Stevens FC, Philipsen H (1997). Assessing the responsiveness of a functional status measure: the Sickness Impact Profile versus the SIP68. J Clin Epidemiol.

[B28] Steiner GL, Norman DR (1996). Health measurement scales: a practical guide to their development and use.

[B29] Hufford MR, Shiffman S (2002). Methodological issues affecting the value of patient-reported outcomes data. Expert Rev Pharmac Outcomes Res.

[B30] Simms RW, Felson DT, Goldenberg DL (1991). Development of preliminary criteria for response to treatment in fibromyalgia syndrome. J Rheumatol.

[B31] Dunkl PR, Taylor AG, McConnell GG, Alfano AP, Conaway MR (2000). Responsiveness of fibromyalgia clinical trial outcome measures. J Rheumatol.

[B32] Heijde DM van der, van't Hof MA, van Riel PL, Theunisse LA, Lubberts EW, van Leeuwen MA, van Rijswijk MH, Putte LB van de (1990). Judging disease activity in clinical practice in rheumatoid arthritis: first step in the development of a disease activity score. Ann Rheum Dis.

[B33] van Gestel AM, Prevoo ML, van't Hof MA, van Rijswijk MH, Putte LB van De, van Riel PL (1996). Development and validation of the European league against rheumatism response criteria for rheumatoid arthritis. Comparison with the preliminary American College of Rheumatology and the World Health Organization/International League Against Rheumatism Criteria. Arthritis Rheum.

[B34] Tugwell P, Boers M, Brooks P, Simon L, Strand V, Idzerda L (2007). OMERACT: an international initiative to improve outcome measurement in rheumatology. Trials.

[B35] Turk DC, Dworkin RH, Allen RR, Bellamy N, Brandenburg N, Carr DB, Cleeland C, Dionne R, Farrar JT, Galer BS, Hewitt DJ, Jadad AR, Katz NP, Kramer LD, Manning DC, McCormick CG, McDermott MP, McGrath P, Quessy S, Rappaport BA, Robinson JP, Royal MA, Simon L, Stauffer JW, Stein W, Tollett J, Witter J (2003). Core outcome domains for chronic pain clinical trials: IMMPACT recommendations. Pain.

[B36] Staud R, Vierck CJ, Robinson ME, Price DD (2006). Overall fibromyalgia pain is predicted by ratings of local pain and pain-related negative affect - possible role of peripheral tissues. Rheumatology.

[B37] Wolfe F (2003). Pain extent and diagnosis: development and validation of the regional pain scale in 12,799 patients with rheumatic disease. J Rheumatol.

[B38] Zautra AJ, Fasman R, Parish BP, Davis MC (2007). Daily fatigue in women with osteoarthritis, rheumatoid arthritis, and fibromyalgia. Pain.

[B39] Wolfe F (1997). The relation between tender points and fibromyalgia symptom variables: evidence that fibromyalgia is not a discrete disorder in the clinic. Ann Rheum Dis.

[B40] Offenbacher M, Glatzeder K, Ackenheil M (1998). Psychological distress and depression in male and female patients with fibromyalgia. Arthritis Rheum.

[B41] Geisser ME, Roth RS, Theisen ME, Robinson ME, Riley JL (2000). Negative affect, self-report of depressive symptoms, and clinical depression: relation to the experience of chronic pain. Clin J Pain.

[B42] Salaffi F, Stancati A, Grassi W (2006). Reliability and validity of the Italian version of the Chronic Pain Grade questionnaire in patients with musculoskeletal disorders. Clin Rheumatol.

[B43] Elliott AM, Smith BH, Penny KI, Smith WC, Chambers WA (1999). The epidemiology of chronic pain in the community. Lancet.

[B44] McBeth J, Macfarlane GJ, Hunt IM, Silman AJ (2001). Risk factors for persistent chronic widespread pain: a community-based study. Rheumatology.

[B45] McBeth J, Macfarlane GJ, Benjamin S, Silman AJ (2001). Features of somatization predict the onset of chronic widespread pain: results of a large population-based study. Arthritis Rheum.

[B46] Nicassio PM, Moxham EG, Schuman CE, Gevirtz RN (2002). The contribution of pain, reported sleep quality, and depressive symptoms to fatigue in fibromyalgia. Pain.

[B47] Schanberg LE, Gil KM, Anthony KK, Yow E, Rochon J (2005). Pain, stiffness, and fatigue in juvenile polyarticular arthritis. Arthritis Rheum.

[B48] Goubert L, Crombez G, De Bourdeaudhuij I (2004). Low back pain, disability and back pain myths in a community sample: prevalence and interrelationships. Eur J Pain.

[B49] Blyth FM, March LM, Brnabic AJ, Jorm LR, Williamson M, Cousins MJ (2001). Chronic pain in Australia: a prevalence study. Pain.

[B50] Rustøen T, Wahl AK, Hanestad BR, Lerdal A, Paul S, Miaskowski C (2004). Prevalence and characteristics of chronic pain in the general Norwegian population. Eur J Pain.

[B51] Callahan LF, Smith WJ, Pincus T (1989). Self-report questionnaires in five rheumatic diseases. Arthritis Care Res.

[B52] Bombardier C, Melfi CA, Paul J, Green R, Hawker G, Wright J, Coyte P (1995). Comparison of a generic and a disease-specific meaure of pain and physical function after knee replacement surgery. Med Care.

[B53] Wolfe F, Rasker JJ (2006). The Symptom Intensity Scale, fibromyalgia, and the meaning of fibromyalgia-like symptoms. J Rheumatol.

